# Where have all the children gone? High HIV prevalence in infants attending nutrition and inpatient entry points

**DOI:** 10.1002/jia2.25089

**Published:** 2018-02-26

**Authors:** Charles Kiyaga, Brittany Urick, Youyi Fong, Christopher Okiira, Nicolette Nabukeera‐Barungi, Denis Nansera, Emmanuel Ochola, Julius Nteziyaremye, Victor Bigira, Isaac Ssewanyana, Peter Olupot‐Olupot, Trevor Peter, Anisa Ghadrshenas, Lara Vojnov

**Affiliations:** ^1^ Central Public Health Laboratories Kampala Uganda; ^2^ Clinton Health Access Initiative Kampala Uganda; ^3^ Fred Hutchinson Cancer Research Center Seattle WA USA; ^4^ Makerere University College of Health Sciences Kampala Uganda; ^5^ Mbabara Regional Referral Hospital Mbarara Uganda; ^6^ Department of HIV, Research and Documentation St. Mary's Hospital Lacor Gulu Uganda; ^7^ Department of Paediatrics/Research Unit Mbale Regional Referral Hospital Busitema University Mbale Uganda

**Keywords:** HIV, infants, entry point, prevalence, case finding

## Abstract

**Introduction:**

Despite notable progress towards PMTCT, only 50% of HIV‐exposed infants in sub‐Saharan Africa were tested within the first 2 months of life and only 30% of HIV‐infected infants are on antiretroviral treatment. This study assessed HIV prevalence in infants and children receiving care at various service entry points in primary healthcare facilities in Uganda.

**Methods:**

A total of 3600 infants up to 24 months of age were systematically enrolled and tested at four regional hospitals across Uganda. Six hundred infants were included and tested from six facility entry points: PMTCT, immunization, inpatient, nutrition, outpatient and community outreach services.

**Findings:**

The traditional EID entry point, PMTCT, had a prevalence of 3.8%, representing 19.6% of the total HIV‐positive infants identified in the study. Fifty percent of the 117 identified HIV‐positive infants were found in the nutrition wards, which had a prevalence of 9.8% (*p* < 0.001 compared to PMTCT). Inpatient wards had a prevalence of 3.5% and yielded 17.9% of the HIV‐positive infants identified. Infants tested at immunization wards and through outreach services identified 0.8% and 1.7% of the HIV‐positive infants respectively, and had a prevalence of less than 0.3%.

**Conclusions:**

Expanding routine early infant diagnosis screening beyond the traditional PMTCT setting to nutrition and inpatient entry points will increase the identification of HIV‐infected infants. Careful reflection for appropriate testing strategies, such as maternal re‐testing to identify new HIV infections and HIV‐exposed infants in need of follow‐up testing and care, at immunization and outreach services should be considered given the expectedly low prevalence rates. These findings may help HIV care programmes significantly expand testing to improve access to early infant diagnosis and paediatric treatment.

## 1 Introduction

Only 51% of HIV‐positive children across 21 high‐burden sub‐Saharan African countries are on lifesaving antiretroviral therapy (ART) 1. Data from this region show that in the absence of treatment, peak mortality for infants infected with HIV *in utero* or *intrapartum* occurs between 2 and 3 months of age 2, and approximately 50% of untreated HIV‐positive infants die by 2 years of age 3. Early initiation on ART for HIV‐positive infants has been shown to significantly reduce mortality 4.

Under the Global Plan Towards the Elimination of New HIV Infections Among Children by 2015 and Keeping Their Mothers Alive, prevention of mother to child transmission (PMTCT) coverage increased significantly between 2009 and 2015, from 37% to 90% 1. Furthermore, 20 of the 21 Global Plan priority high‐burden countries currently offer lifelong ART to pregnant women (WHO Option B+) rather than time‐limited prophylaxis (WHO Option A or Option B). As a result, over 90% of pregnant women who access ART for PMTCT have access to lifelong therapy 1. Similarly, in Uganda where the HIV prevalence rate among pregnant mothers is 7.6%, approximately 85% of newly diagnosed pregnant mothers were initiated on ART in 2016. Conversely, the increase in ART coverage amongst children over the same time period, from 10% in 2009 to 35% in 2015, was significantly lower and overall ART coverage among infants is less than 50% of maternal coverage. This is largely because HIV‐exposed infants either do not receive a timely, actionable result or are not tested at all. In 2015, only 51% of HIV‐exposed infants received a virological test by 2 months of age, the WHO‐recommended time period for a first early infant diagnosis (EID) test 1. Improvements in EID coverage have been minimal in recent years and some countries have reported testing plateaus or declines 1.

Doubling down on efforts to find HIV‐exposed and HIV‐positive children is necessary. Evidence demonstrates that in many contexts relying solely on PMTCT programmes to achieve universal EID coverage is inadequate and paediatric ART coverage stagnates without strengthened diagnostic programmes targeting children 5, 6. To make a significant shift from the status quo, a comprehensive range of infant testing strategies specific to both high and low prevalence settings – and a clear articulation of how to implement them – are needed.

To date, provider‐initiated HIV testing and counseling (PITC) has been recommended by WHO as a mechanism to identify HIV‐exposed infants who are lost to follow up from or never enrolled in the PMTCT cascade. Indeed, many countries have already incorporated paediatric PITC recommendations into national guidelines 7, 8. However, persistently low EID coverage – which translates into low paediatric ART coverage – suggests that despite the inclusion of paediatric PITC in policy, uptake and practice are insufficient.

Several studies have examined the implementation of PITC or routine testing at different healthcare facility entry points in isolation, but to date no studies have compared HIV prevalence at multiple entry points simultaneously. Many of these studies also lack a comparator, such as the traditional PMTCT entry point 9, 10, 11, 12, 13. Furthermore, many do not employ random, systematic, or consecutive sampling, but rather targeted testing, which means that the underlying prevalence for individual entry points is difficult to ascertain 9, 10. Therefore, this study was conducted to determine and compare paediatric HIV prevalence at several healthcare facility entry points in order to better understand how to identify more HIV‐positive children in resource‐limited settings.

## 2 Methods

This was a cross‐sectional prospective study of infants below the age of 2 years presenting at the primary healthcare facility entry points at four hospitals in Uganda. The four healthcare facilities included in this study were: Mulago National Referral Hospital; St. Mary's Hospital, Lacor; Mbale Regional Referral Hospital; and Mbarara Regional Referral Hospital. Infants were recruited from six entry points at each healthcare facility: Immunization (Expanded Programme on Immunization)/well‐child clinic, paediatric outpatient, paediatric inpatient, nutrition, outreach and PMTCT, which is the traditional setting for EID testing. A total of 3600 infants less than 2 years of age were included in the study. One hundred and fifty infants were enrolled at each of the six entry points per hospital. Data collection occurred between September 2014 and August 2015.

Patients were systematically sampled and enrolled at each entry point. Due to low daily volumes (<16 infants/day), consecutive enrolment was employed at the nutrition and PMTCT entry points. Systematic sampling across all attending patients was used within the immunization, paediatric outpatient, paediatric inpatient and outreach settings due to high patient volume, to ensure unbiased patient selection. It was predicted that each entry point could enrol 15 infants per day per study nurse; therefore, if an entry point typically had 16 to 30, 31 to 45, or 46 to 60 eligible infants per day each study nurse would enrol every other, third, or fourth infant respectively. Study systems were put in place to ensure no infant was enrolled at multiple entry points. The study objectives and study enrolment processes, including pre‐HIV test counselling, were explained to the mother or guardian of each infant invited to participate in the study at non‐PMTCT entry points before they signed a letter of informed consent.

Demographic and clinical data were collected for each infant and mother (if present) using standardized study‐specific forms and study‐specific identification numbers. All enrolled infants underwent both serological and virological testing to determine HIV exposure and HIV infection status respectively. Dried blood spot specimens (DBS) were collected for virological testing and rapid serological diagnostic tests were conducted on each patient. Both tests were conducted on each enrolled infant regardless of the respective results, except for infants at the PMTCT entry point who did not receive a serological screen as their exposure status was already known. Healthcare facility staff, including nurses, clinical officers and laboratory technicians, were trained on study procedures, how to conduct DBS specimen collection and rapid diagnostic testing, and demonstrated proficiency before study commencement. Finally, maternal HIV status was determined if she provided verbal identification or by confirmation from facility records, if her infant included in the study was HIV‐positive, or if her infant was positive by RDT. All mothers were offered HIV testing per the national guidelines.

Rapid diagnostic testing was performed using the Alere Determine™ HIV‐1/2 (Waltham, MA, USA). One drop of whole blood was collected using a lancet heel stick, applied to the test strip, and tested per manufacturer's instructions. Either that same lancet heel stick or a fresh draw was used to collect an additional 3 to 5 drops of whole blood that were applied to a filter paper card (Whatman 903, GE Healthcare Biosciences, Pittsburgh, PA). Specimens were dried overnight at room temperature and shipped weekly for testing to the Central Public Health Laboratories in Kampala, Uganda. Dried blood spot specimens were processed and tested with the Roche COBAS AmpliPrep/COBAS TaqMan (CAP/CTM 96) HIV‐1 Qualitative Test (Roche Molecular Diagnostics, Branchburg, NJ, USA) according to the manufacturer's instructions.

Any infants with positive rapid diagnostic test or virological test result were referred to PMTCT for post‐test counselling and inclusion in care and treatment per the national standard of care guidelines.

This study was approved by the Mildmay Uganda Research Ethics Committee, Uganda National Council for Science and Technology, Mulago Hospital Research and Ethics Committee, Institutional Review Committee at St. Mary's Hospital, Lacor, and the Chesapeake Institutional Review Board in the USA.

Statistical analysis was performed with the R statistical software (Version 3.3.2, Free Software Foundation, Boston, MA, USA) and GraphPad Prism (Version 6.0, La Jolla, CA, USA). Infants from like entry points were pooled across hospitals for primary analyses. Two‐sample and multi‐sample comparisons were done using the nonparametric rank‐based Wilcoxon‐Mann–Whitney and Kruskal–Wallis tests respectively. Multi‐testing *p*‐value adjustment for the *p* values from logistic regression models was performed according to Hothorn *et al*. 14 and controls family‐wise error rate. Binomial probability confidence interval was computed using the Wilson method 15.

## 3 Results

A total of 3600 infants were enrolled (47% female) (Table 1). Just over half of the infants included were 8 months of age or younger (56%) and 74% of infants were 12 months of age or younger. The median age at study inclusion was 7 months (IQR: 2 to 13 months). In the HIV‐positive study population, 40% of infants were 8 months of age or younger, and 57% were 12 months of age or younger. The median age at study inclusion for HIV‐positive infants was 11 months (IQR: 5 to 15 months). Most (80%) infants were breastfeeding at the time of testing; however, only 46% of the HIV‐positive infants were breastfeeding at the time of testing (*p* < 0.001). Approximately 50% of infants had attended a healthcare facility at some point within the previous year in all study groups.

**Table 1:** Demographic characteristics of study participants

	Total, N = 3,600	Total, N = 3,481	Total, N = 117
	All infants	HIV‐negative infants	HIV‐positive infants
Gender, n (%)			
Female	1,691 (47)	1,638 (47)	52 (44)
Male	1,909 (53)	1,843 (53)	65 (55)
Age group, n (%)			
0 to 4 months	1346 (37)	1317 (38)	28 (24)
4 to 8 months	666 (18)	647 (19)	19 (16)
8 to 12 months	643 (18)	622 (18)	21 (18)
12 to 16 months	448 (12)	424 (12)	23 (20)
16 to 20 months	291 (8)	279 (8)	12 (10)
20 to 24 months	206 (6)	192 (6)	14 (12)
Median age, months (IQR)			
PMTCT	1 (1 to 2)	1 (1 to 2)	4 (2 to 10)
Nutrition	12 (8 to 17)	12 (8 to 17)	13 (7 to 17)
Inpatient	9 (5 to 14)	9 (5 to 14)	13 (5 to 15)
Outpatient	9 (5 to 15)	9 (5 to 15)	8 (6 to 14)
Immunization	4 (2 to 9)	4 (2 to 9)	1 (1 to 1)
Outreach	8 (4 to 13)	8 (4 to 13)	20 (19 to 22)
Total	7 (2 to 13)	7 (2 to 13)	11 (5 to 15)
Breastfeeding status, n (%)			
Currently breastfeeding	2841 (79)	2784 (80)	55 (47)
Not breastfeeding	737 (20)	677 (19)	60 (51)
Unknown	22 (1)	20 (1)	2 (2)
Facility attendance within previous year, n (%)			
Attended	1741 (48)	1676 (48)	64 (55)
Did not attend	1227 (34)	1197 (34)	30 (26)
N/A	632 (18)	608 (17)	23 (2)

**Figure 1:**
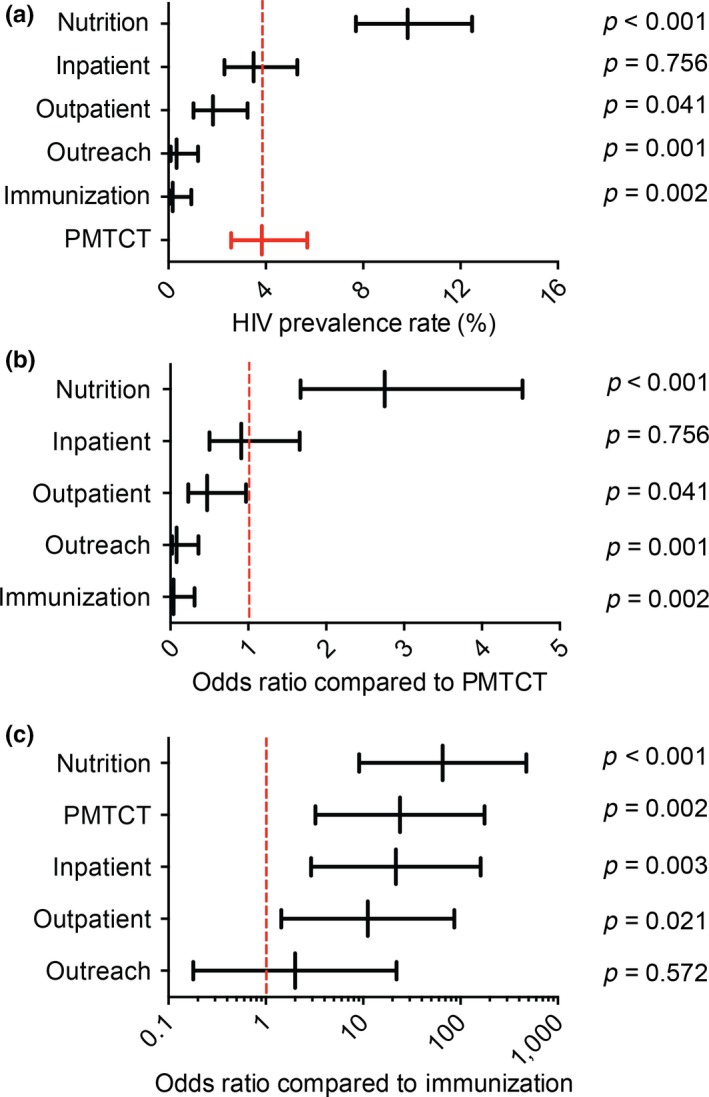
HIV prevalence proportions and odds ratios across facility entry points. (a) HIV prevalence in each facility entry point. (b) Odds ratios to identify an HIV‐positive infant in each facility entry point compared to the traditional PMTCT setting. (c) Odds ratios to identify an HIV‐positive infant in each facility entry point compared to the immunization entry point.

Of the 3600 infants enrolled in the study, two were excluded from additional analyses. One infant from the immunization entry point had an invalid (failed) virological test result, and one infant from the PMTCT entry point did not have a recorded virological test result. A total of 117 (3.3%, 95% CI: 2.7% to 3.9%) infants were identified as HIV‐positive. Infants tested at the traditional PMTCT entry point had a prevalence of 3.8% (95% CI: 2.6% to 5.7%), yielding approximately 20% of all identified HIV‐positive infants (Table 2, Figure 1a). Fifty percent of the 117 HIV‐positive infants identified in this study were found at the nutrition entry point, which had a prevalence of 9.8% (95% CI: 7.7% to 12.5%) (*p* < 0.001, compared to PMTCT). The inpatient entry point had a similar yield of HIV‐positive infants (18%) as the PMTCT entry point and a prevalence of 3.5% (95% CI: 2.3 to 5.3). While the prevalence at the outpatient entry point was relatively modest at 1.8% (95% CI: 1.0% to 3.3%), 11 HIV‐positive infants or 9.4% of all identified HIV‐positive infants, were identified. The immunization and community outreach entry points had the lowest prevalence at less than 0.3% and also the smallest number of HIV‐positive infants were identified, one and two respectively.

**Table 2:** Number and prevalence of HIV to positive infants by entry point and facility

	PMTCT	Immunization	Inpatient	Nutrition	Outpatient	Outreach	Total
Number	23	1	21	59	11	2	117
Prevalence (95% CI)	3.8% (2.6 to 5.7)	0.2% (0.0 to 0.9)	3.5% (2.3 to 5.3)	9.8% (7.7 to 12.5)	1.8% (1.0 to 3.3)	0.3% (0.1 to 1.2)	3.3% (2.7 to 3.9)
Lacor	6.0%	0.0%	0.0%	4.0%	0.7%	0.7%	1.9% (1.2 to 3.0)
Mbale	1.3%	0.7%	0.7%	13.3%	2.0%	0.7%	3.1% (2.2 to 4.5)
Mbarara	4.7%	0.0%	6.7%	11.3%	2.7%	0.0%	4.2% (3.1 to 5.7)
Mulago	3.3%	0.0%	6.7%	10.7%	2.0%	0.0%	3.8% (2.7 to 5.2)

The overall prevalence of HIV‐positive infants was consistent across facilities (3.1% to 4.2%) besides Lacor, which had a lower prevalence (1.9%). The number and proportion of HIV‐positive infants was generally lower at Lacor across most entry points, particularly the nutrition entry point (*p* < 0.001).

The odds ratios for identifying an HIV‐positive infant at each entry point were calculated as compared to the traditional PMTCT entry point while adjusting for facilities using a logistic regression model (Figure 1b). Only the nutrition entry point had an odds ratio greater than one (2.8; 95% CI: 1.7 to 4.5; *p* < 0.001), indicating that the infants attended nutrition entry points were three times more likely to be HIV‐positive than those at PMTCT. The inpatient entry point had an odds ratio of 0.9 (95% CI: 0.5 to 1.7; *p* = 0.756). The outpatient entry point had an odds ratio of 0.5 (95% CI: 0.2 to 1.0; *p* = 0.041). The outreach and immunization entry points had odds ratios of 0.1 (95% CI: 0.0 to 0.4; *p* = 0.001) and 0.0 (95% CI: 0.0 to 0.3; *p* = 0.002) respectively, indicating significantly lower likelihood of infants at outreach and immunization being HIV‐positive compared to PMTCT.

Mechanisms to leverage the Expanded Programme on Immunization as a setting to provide access to EID are frequently discussed in the global paediatric HIV community. We, therefore, also determined the odds of identifying an HIV‐positive infant at each entry point compared to the immunization entry point (Figure 1c). The nutrition, PMTCT, and inpatient entry points had odds ratios of 65.7 (95% CI: 9.1 to 474.7; *p* < 0.001), 23.9 (95% CI: 3.2 to 177.3; *p* = 0.002), and 21.7 (95% CI: 2.9 to 161.7; *p* = 0.003) respectively, indicating that each would be significantly more efficient to identify HIV‐positive infants compared to the immunization entry point. The odds of identifying HIV‐positive infants at outpatient and outreach entry points were also higher than at immunization clinics, 11.2 (95% CI: 1.4 to 86.7; *p* = 0.021) and 2.0 (95% CI: 0.2 to 22.1; *p* = 0.572) respectively.

The proportion of HIV‐positive infants (and mothers) identified at non‐PMTCT entry points who were previously enrolled in PMTCT was determined in order to understand whether HIV‐positive infants identified in the study were missed in the traditional EID setting. A mother was identified as HIV‐positive if she provided verbal identification or by confirmation from facility records, if her infant included in the study was HIV‐positive, or if her infant was positive by RDT. By these three definitions, 324 mothers were HIV‐positive for a total of 325 HIV‐exposed infants (one set of twins). Only 60% of all HIV‐positive mothers were enrolled in PMTCT (Table 3). Most (80%) HIV‐positive mothers knew their HIV status, and 74.9% of those mothers were enrolled in PMTCT. Of those HIV‐positive mothers with an HIV‐positive infant, only 18.1% were enrolled in PMTCT. Forty‐seven percent of HIV‐positive mothers with an HIV‐positive infant knew their status, and of those only 38.7% were enrolled in PMTCT.

**Table 3:** PMTCT characteristics of HIV‐positive mother study participants

	Total, N = 324 (100%)	Total, N = 94 (29%)	Total, N = 230 (71%)
	All HIV‐positive mothers, n (%)	HIV‐positive mothers with HIV‐positive infants, n (%)	HIV‐positive mothers with HIV‐negative infants, n (%)
Mother's knowledge of own status			
Self‐identified positive	259 (79.9)	44 (46.8)	215 (93.5)
Self‐identified negative	19 (5.9)	15 (16.0)	4 (1.7)
Self‐identified unknown	46 (14.2)	35 (37.2)	11 (4.8)
Mother‐infant pair enrolled in PMTCT			
Yes	194 (59.9)	17 (18.1)	177 (77.0)
No	130 (40.1)	77 (81.9)	53 (23.0)

## 4 Discussion

While previous studies provided clues to the highest impact entry points for identifying HIV‐positive infants 9, 10, 11, 12, 13, these findings present the first evidence of differences in HIV prevalence amongst children accessing care at different health facility entry points in a low resource, generalized HIV epidemic. These data demonstrate high HIV prevalence among infants presenting for care at nutrition, inpatient, and PMTCT entry points. In particular, prevalence at nutrition wards was over two times higher than in the routine PMTCT setting. HIV prevalence was much lower at the immunization and outreach entry points; the likelihood of identifying a HIV‐positive infant was more than 20 times greater in nutrition and inpatient wards than in immunization clinics. These results suggest a comprehensive strategy for expanded routine EID screening at entry points beyond PMTCT that can help maximize the identification of HIV‐positive infants in resource‐limited settings.

In generalized epidemic settings, the World Health Organization (WHO) strongly recommends that infants and children with unknown HIV status who are admitted at inpatient or nutrition entry points should be routinely tested for HIV. Furthermore, WHO conditionally recommends offering testing in outpatient or immunization entry points 16, 17. The findings of this study align with these WHO recommendations. However, while these policies already exist in many high burden countries 8, they are not widely put into practice and PMTCT remains the primary testing point for early infant diagnosis. Up to 23% of mother‐infant pairs do not access PMTCT in high HIV burden countries and significant proportions are lost‐to‐follow‐up during EID testing, pre‐ART as well as after ART initiation 18, 19. These infants may subsequently present at nutrition and inpatient wards, as a result of the significant morbidity amongst untreated HIV‐positive infants 2, 3. As countries seek to expand the coverage of paediatric treatment, this study highlights the importance of implementing more diverse testing strategies outside of PMTCT to expand access to EID and reduce the current gaps in access to paediatric HIV care.

Our results support universal testing of infants in nutrition and inpatient entry points within generalized epidemic settings. While widespread virological testing of all infants who present for care at healthcare facilities may be difficult to implement or unnecessary in every setting, universal testing at nutrition and inpatient entry points in high prevalence settings may play a key role in expanding paediatric ART access. In this context, the use of point‐of‐care EID technologies in these settings, as logistics and budgets warrant, to allow for immediate testing, same‐day return of results, and rapid clinical decision‐making is a potential opportunity or tool for health programmes to consider. Previous evidence suggests that rapid EID testing leads to faster ART initiation 20, while earlier ART initiation can reduce infant mortality 4.

Universal testing in lower‐yield settings, such as immunization and community outreach entry points, may not be as efficient in identifying HIV‐positive infants, but alternative strategies for identifying high‐risk infants could be pursued. Previous studies have investigated the acceptability and feasibility of expanding EID testing services outside of PMTCT – including community outreach, door‐to‐door testing, and immunization settings 11. Vaccination rates are high across many countries, and immunization is an entry point where significant numbers of infants present. While screening of all infants at immunization has been shown to be feasible 11, 21, the results of this study suggest that universal EID testing at immunization and outreach may be relatively inefficient due to the low prevalence of HIV‐positive infants. Earlier studies in Malawi and Nigeria presented similar low prevalence of HIV‐positive infants at immunization 10, 22. Furthermore, the complexity and cost of integrating universal EID into congested immunization clinics has been previously noted 11, 23 and may not be an effective use of limited resources. Immunization and outreach entry points could, however, be ideal settings for maternal re‐testing and symptom‐driven screening to identify HIV‐exposed infants for referral into paediatric HIV care 24.

Of the HIV‐positive infants in the study the median age was almost 1 year, and over 50% had previously attended a healthcare facility, suggesting that opportunities to identify either the HIV‐infected mothers or their infants earlier were missed. Option B+, lifelong ART for HIV‐positive pregnant women, was adopted in Uganda in 2012 and has since achieved national coverage. In 2014, Uganda reported >95% ART coverage for its PMTCT population, thus exceeding the Global Plan target of 90%, and the mother‐to‐child transmission rate was 4.2% 1. In this study, we found that 75% of all HIV‐positive mothers identified in non‐PMTCT entry points who knew their status had enrolled in PMTCT. Understanding the reasons for the gap between status knowledge and willingness, interest, or ability to enrol in PMTCT will be critical to further reducing mother‐to‐child transmission. This gap was particularly large in the study population of HIV‐positive mothers who had HIV‐positive infants; of the mothers in this population who knew their HIV‐positive status, less than 40% had enrolled in PMTCT.

Furthermore, maternal re‐testing should be more strongly emphasized, particularly during breastfeeding and postnatal follow‐up. Approximately 20% of all HIV‐positive mothers in the study identified as HIV‐negative or did not know their status, and over 50% of HIV‐positive mothers with an HIV‐positive infant identified as HIV‐negative or did not know their status. Finally, we found that 8.8% of mothers enrolled in PMTCT had an HIV‐positive infant, which is generally higher than previous studies in Option B+ settings 25, 26, 27, 28, 29. Although not nationally representative, these data shed important light on the ongoing challenges in the fight to eliminate mother‐to‐child transmission and suggest a need to strengthen each step in the PMTCT cascade in order to achieve ambitious elimination goals 30.

Several limitations exist within this study. The study was not designed as a nationally representative generalizable survey; however, healthcare facilities were selected from each of the four major geographies of Uganda. Mbarara and Mbale Regional Referral Hospitals and Mulago National Referral Hospital had similar HIV‐positive case numbers and prevalence for each entry point indicating that the data are not isolated to specific regions or facilities; however, Lacor had lower prevalence at most entry points, comparatively. We suspect that this may have been due to higher PMTCT and Option B+ coverage at Lacor. These differences could account for the variability in the prevalence observed across the healthcare facilities. In addition, only high volume healthcare facilities with distinct entry points were included to allow for EID testing volumes to be comparable to the infant volumes at other entry points and to clearly address our proposed hypothesis with an efficient approach. Though the results cannot immediately be extrapolated to smaller healthcare facilities, it is possible that these findings can be applied to smaller healthcare facilities through thoughtful implementation considerations. Infants suspected of malnutrition or in need of referral to inpatient facilities could be prioritized for routine testing. Finally, due to the robust patient selection process, limited bias could be expected. However, we acknowledge that our study was primarily limited to facility‐based services, besides the outreach entry point, and included only those infants who presented to the entry points examined.

As low‐resource, high HIV burden countries continue to face funding challenges, national government and global policymakers are seeking cost‐effective, high impact interventions to reach the UNAIDS 90‐90‐90 targets 31. National EID programmes across sub‐Saharan Africa are currently not providing access to EID testing to a significant proportion of HIV‐exposed infants. Universal virological testing using conventional laboratory‐based or point‐of‐care EID testing at nutrition, inpatient and outpatient entry points should, therefore, be prioritized and implemented as routine practice at all healthcare facilities.

## 5 Conclusions

The HIV prevalence at the nutrition and inpatient wards were 9.8% and 3.5% (*p* < 0.001 compared to PMTCT). Expanding routine early infant diagnosis screening beyond the traditional PMTCT setting to nutrition and inpatient entry points will increase the identification of HIV‐infected infants. Furthermore, maternal re‐testing at immunization, outreach and during postnatal follow‐up may help identify those mother‐baby pairs missed as well as incident maternal infections. These findings may help HIV care programmes significantly expand testing to improve access to early infant diagnosis and paediatric treatment.

## Competing interests

All authors declare no conflict of interest.

## Authors' contributions

CK, BU, NB, DN, EO, JN, POO, AG and LV developed the protocol including literature search and study design. BU, CO, VB and IS were involved in data collection. CK, BU, YF, IS, TP, AG and LV were involved in data analysis. All authors contributed to editing the final report, were involved in the development of the primary manuscript, interpretation of data, and read and approved the final version.

## Ethics committee approval

This study was approved by the Mildmay Uganda Research Ethics Committee, Uganda National Council for Science and Technology, Mulago Hospital Research and Ethics Committee, Institutional Review Committee at St. Mary's Hospital, Lacor, and the Chesapeake Institutional Review Board in the USA.

## Funding

The funding for this study was from the United Kingdom's Department for International Development (DFID). No funding bodies had any role in study design, data collection and analysis, decision to publish, or preparation of the manuscript.
